# Supplementary material for: From sequence to enzyme mechanism using multi-label machine learning
Source: BMC Bioinformatics. 2014 May 19;15:150. doi: 10.1186/1471-2105-15-150 (PMC4229970; doi:10.1186/1471-2105-15-150)
Supplement: Additional file 2 — Java code of ml2db. Additional file ml2db_code.tar.gz contains the Java source code to run the multi-label machine learning experiments and save the results to database. The code’s Javadoc is included. [file 1471-2105-15-150-S2.zip › additional file 2/ml2db/ml2db/doc/main/ml2db/al/utility/hybrid/package-summary.html]

main.ml2db.al.utility.hybrid


JavaScript is disabled on your browser.


- Overview
- Package
- Class
- Use
- Tree
- Deprecated
- Index
- Help

- Prev Package
- Next Package

- Frames
- No Frames

- All Classes

# Package main.ml2db.al.utility.hybrid

- Class Summary

  | Class | Description |
  | --- | --- |
  |  |  |
  | --- | --- |
  | GlobalDistanceWithConfidenceMetric | Combines a Positioning metric (a metric that considers positively if an instance is close to other unlabelled instances and distant from other labelled instances) with the average certainty of the instance prediction (that is, how distant the average of confidence over all labels is from 50%) |

- Overview
- Package
- Class
- Use
- Tree
- Deprecated
- Index
- Help

- Prev Package
- Next Package

- Frames
- No Frames

- All Classes
